# Silencing of the MEG3 gene promoted anti‐cancer activity and drug sensitivity in glioma

**DOI:** 10.1111/jcmm.17883

**Published:** 2023-07-31

**Authors:** Zehra Degirmenci, Sena Unver, Turker Kilic, Timucin Avsar

**Affiliations:** ^1^ Neuroscience Laboratory Health Sciences Institute, Bahcesehir University Istanbul Turkey; ^2^ Department of Neurosurgery Bahcesehir University School of Medicine Istanbul Turkey; ^3^ Department of Medical Biology Bahcesehir University School of Medicine Istanbul Turkey

**Keywords:** cell migration, cell proliferation, drug sensitivity, glioma, MEG3

## Abstract

Aberrant expression of MEG3 has been shown in various cancers. The purpose of this study is to evaluate the effect of MEG3 on glioma cells and the use of potential chemotherapeutics in glioma by modulating MEG3 expression. Cell viability, migration and chemosensitivity were assayed. Cell death was evaluated in MEG3 overexpressing and MEG3 suppressed cells. MEG3 expression was compared in patient‐derived glioma cells concerning IDH1 mutation and WHO grades. Silencing of MEG3 inhibited cell proliferation and reduced cell migration while overexpression of MEG3 promoted proliferation in glioma cells. MEG3 inhibition improved the chemosensitivity of glioma cells to 5‐fluorouracil (5FU) but not to navitoclax. On the other hand, there is no significant effect of MEG3 expression on temozolamide (TMZ) treatment which is a standard chemotherapeutic agent in glioma. Suppression of the MEG3 gene in patient‐derived oligodendroglioma cells also showed the same effect whereas glioblastoma cell proliferation and chemosensitivity were not affected by MEG3 inhibition. Further, as a possible cell death mechanism of action apoptosis was investigated. Although MEG3 is a widely known tumour suppressor gene and its loss is associated with several cancer types, here we reported that MEG3 inhibition can be used for improving the efficiency of known chemotherapeutic drug sensitivity. We propose that the level of MEG3 should be evaluated in the treatment of different glioma subtypes that are resistant to effective drugs to increase the potential effective drug applications.

## 1 INTRODUCTION

Treatment modalities for glioblastoma are limited due to the complex nature and malignant progression of the tumour. In recent years, efforts to develop novel therapeutic strategies for efficient treatments in glioma cells focused on inhibiting or activating essential regulatory genes.^1^, ^2^ Understanding the role of non‐coding genes in glioma may lead us to discover novel therapeutic targets. Non‐coding genes make up the vast majority of the human genome. MicroRNAs (miRNA) and long non‐coding RNAs (lncRNA) are expressed differently in cancer cells than in healthy cells. Noncoding RNAs regulate the basic characteristics of cancer cells such as cell proliferation, survival, invasion and therapy resistance. The functions of lncRNAs emerge through epigenetic, transcriptional and post‐transcriptional mechanisms which have not yet been fully understood. Understanding the role of lncRNAs in these mechanisms will help us in the treatment of glial tumours as well as many types of cancer.^3^, ^4^, ^5^

Maternally expressed gene 3 (MEG3) is an imprinted gene residing in chromosome 14 which also hosts long non‐coding RNAs, microRNAs, and nucleolar RNAs. Hence, it plays several vital roles in growth and development. A defect in the expression of MEG3 can lead to various diseases including cancer as a result of the disruption caused in the regulation of cellular proliferation and its putative role as a tumour suppressor. Several studies showed that MEG3 played various roles in different cancer types. Overexpression of MEG3 was found to inhibit EMT and result in a reduction of the migration and invasion of cervical cancer cells. Whereas in hepatocellular carcinoma, MEG3 was found to contribute to the EMT phenotype and therefore increased the migration and invasion of the cancer cells. Its role may vary depending on the type of cancer.^6^ The most prominent role of MEG3 in glioma is cell cycle regulation. MEG3 overexpression causes the cell cycle to stop in the G2/M phase, thus reducing the proliferation of glioma cells. MEG3 can also upregulate tumour suppressors by interacting with regulatory miRNAs. This interaction is also required for the activation of p53.^7^

The precise role of MEG3 in glioma is still not well understood due to confusing results in previous studies. This study aims to discover the role of MEG3 in glioma cells and to investigate how MEG3 affects cell proliferation, migration and chemosensitivity in glial tumours when overexpressed by the transfection of the MEG3 gene and suppressed by small interference RNA (siRNA). Although, neither navitoclax (Nvtx) nor 5‐fluorouracil (5FU) are available for standard glioma treatment, the aim of this study was to sensitize glioma cells to drugs currently in use and approved for chemotherapeutic activity and to evaluate their use in the treatment of glioma by modulating the MEG3 gene expression. We wanted to test the drugs Nvtx and 5FU because their mode of action in anti‐cancer activity is not the main cellular pathway to be targeted in gliomagenesis (Bcl‐2 inhibitor and cytotoxic agent). Therefore, we aimed to adapt a new inhibitor to be used in the treatment of glioma. Furthermore, the effects of MEG3 gene expression were compared between non‐cancerous human umbilical vein endothelial cord (HUVEC) cells and U87MG glioblastoma cells either derived from ATCC or patients with different pathological grades and histopathological subtypes of glioma tumours.

## 2 METHODS

### 2.1 Cell culture

U87MG and HUVEC cells were obtained from American‐Type Culture Collection (ATCC, USA). Patient‐derived tumour tissues were obtained through surgical operation upon the consent of the patients and with approval from the institutional ethics board (BAU‐2020/02). Primary tumour cells were incubated under the appropriate conditions and regularly passaged. Cell lines were grown in Dulbecco's modified Eagle medium (DMEM) (Wisent, #319‐005CL) supplemented with 1% antibiotic, antimycotic (Wisent, #450‐115EL) and 10% foetal bovine serum (ThermoFisher, #10500064). Whereas primary cells were grown in DMEM mixed with nutrient mixture F‐12 (DMEM/F‐12) (Capricorn, #DMEM‐12A) supplemented with 1% antibiotic, antimycotic (Wisent, #450‐115EL) and 10% foetal bovine serum (ThermoFisher, #10500064). All cells were incubated in a humidified air incubator (5% CO_2_) at 37°C. In cell culture studies cells were seeded at a maximum of 10% density of the wells and sampling (RNA or DNA isolation) was done at a maximum of 80% density except for wound healing assay.

Different groups in this study were formed by transfection of cells with either MEG3 siRNA or overexpression (OE) plasmid and treatment of cells with different drugs either Nvtx (MedChemExpress, #HY‐10087) or 5FU (US Biological, #F5275‐45). Cell culture experiments with cell lines were triplicated and repeated at least two times, therefore *n* = 6 for each cell culture experiment group. Analyses with patient‐derived primary cell studies were triplicated but could not be repeated due to the limited culturing potential of primary cells. The sample size was determined based on the recommendation in the article by Lazic SE.^8^

### 2.2 Cell transfection

The overexpression plasmid pCI‐MEG3 (Addgene, #44727) which was generously gifted to Addgene by Yunli Zhou and colleagues was obtained.^9^ BLOCK‐iT™ RNAi Designer web tool was used to design siRNA against the mRNA of the MEG3 gene. The design was based on the transcript variant 1 of MEG3 and the MEG3 gene sequence was obtained from the NCBI Gene database. One of the three different siRNA sequences suggested by the BLOCK‐iT™ RNAi Designer web tool was selected and ordered from Thermo Fisher (Thermo Fisher, #Stealth 130). The MEG3 siRNA sense sequence was CAUCAUCCGUCCACCUCCUUGUCUU and the MEG3 siRNA antisense sequence was AAGACAAGGAGGUGGACGGAUGAUG. 1,000,000 cells/well were transfected with the MEG3 overexpression plasmid, which was introduced to the cells with the PEI (Serva, #3314104) chemical transfection method. Polyethylenimine (PEI) (1 mg/mL), 500 μL of DMEM medium and 7.5 μg of plasmid DNA were used for transfection. PEI was given the day after the cells were seeded and the cells were harvested for RNA and protein isolation 48 h after transfection. siRNA transfection was done using the Neon™ Transfection System 100 μL kit protocol with 30 nM oligonucleotide. About 1300 V were applied to the samples for 30 ms and 1 pulse. siRNA was given while the cells were seeded, and the cells were collected for RNA and protein isolation after 72 h of incubation for qPCR studies.

### 2.3 Quantitative real‐time PCR (qPCR)

RNA was isolated with the High Pure RNA isolation kit (Roche, #11828665001), and the extracted RNA (100 ng) was reverse transcribed into cDNA with the A.B.T.™ cDNA Synthesis Kit (dT20, C01‐01‐25) The relative expression levels of MEG3 were calculated using the $2^{-∆∆C_{t}}$ method. GAPDH was used as an internal control in the qPCR assays. The primers used are as follows: MEG3 Forward: GCCTGCTGCCCATCTACAC, MEG3 Reverse: CCTCTTCATCCTTTGCCATC, GAPDH Forward: TGCACCACCAACTGCTTAGC and GAPDH Reverse: GGCATGGACTGTGGTCATGAG.

### 2.4 Cell proliferation assay

Cell proliferation and viability were measured with 3‐(4,5‐dimethylthiazol‐2‐yl)‐2,5‐diphenyl‐2H‐tetrazolium bromide (MTT) (US Biological, #258093) assay. 10,000 cells/well were seeded into each well, and the next day 10 μL of MTT solution (5 mg/mL in PBS) was added to each well and incubated for 2.5 h in the incubator. 0.1 M HCl and 10% SDS solubilization buffer were added to dissolve the formazan crystals and incubated for 15 min at 37°C. The absorbance was measured at 570 nm. Maximum cell density at proliferation assay was 80% at 96 h of incubation.

A chemosensitivity assay was performed by administration of two different drugs 5FU and Nvtx. Cell viability upon the administration of these drugs was measured via the MTT assay. 5FU is a common cytotoxic drug that acts as a pyrimidine analog. It has several uses in different cancer types.^10^ Nvtx is another anti‐cancer agent that acts as a Bcl‐2 inhibitor, especially in myeloid chronic lymphocytic leukaemia.^11^ Although both drugs are previously approved for other cancers none of them are used in the standard treatment of glioma tumours. The purpose of using these drugs was to increase glioma cell sensitivity to other drugs. Cells were treated with 100 μM 5FU, and 100 μM Nvtx. The drugs were administered to the cells after the transfection of the siRNA and overexpression plasmids for 96 h.

### 2.5 Wound‐healing assay

Cells were seeded into 12‐well plates with each well containing 30,000 cells/well. After 90% cell confluence was achieved, a scratch was created in the middle of the wells with the help of a pipette tip. Microscope images (Leica DM 2500) of the healing process were obtained until the wound in the control group cells was closed. All groups were separately wounded on three different wells and a single photograph (40×) of each well was taken for each day. Wound Healing Size Tool in ImageJ Software was utilized to quantify the changes in scratch areas. Images were analysed and compared concerning the number of cells migrating to the scratched area on the dish surface using Image J.

### 2.6 Annexin V/Propidium iodide assay

Control and transfected cells were removed with Accutase (Biolegend, #423201) and then collected. Cells were washed twice with PBS and sodium azide (Sigma, #26628–22‐8) solution. Cells were then resuspended in the binding buffer included in the APC Annexin V Apoptosis Detection Kit with PI (Biolegend #640932). 5 μL APC Annexin V was added into the test tube containing the suspended cells. Then 10 μL propidium iodide solution was added. This mixture was gently vortexed, then incubated for 15 min at room temperature and in the dark. Finally, 400 μL binding buffer was added to each tube. The ratio of early apoptotic, late apoptotic and dead cells was measured with flow cytometry (Acea NovoCyte 3005, Thermo).

### 2.7 Primary cell lines and tumour samples

Primary cells were prepared using fresh tumour tissues in experimental studies. The tumour tissue was shredded with sterile scalpels and washed with PBS. The blood vessels are cleaned from the tissue as much as possible and the tissue is taken into the falcon tube. An excess amount of DMEM medium was added to it and incubated until the tissues collapse. The supernatant was removed, the medium was added again and the falcon is centrifuged at 200 ***g*** for 5 min. One oligodendroglioma (OG) (Grade 3 and IDH mutant) and one glioblastoma (GBM) tissue (Grade 4 and IDH wild type) were used for primary cell establishment.

Relative MEG3 RNA level comparison analysis between different glioma tumours was done by qPCR analysis. Tumour samples were obtained from Bahcesehir University Brain Tumour Tissue Bank upon institutional ethics committee approval (BAU‐2020/02). Tumour tissues were randomly chosen from the tissue bank and the experimenter was blind to samples. A total of 30 samples with 6 pilocytic astrocytomas (Grade 1), 5 astrocytomas (Grade 2), 9 oligodendroglioma (Grade 3) and 10 glioblastomas (Grade 4) were included in the study. The mean age of patients was 43.11 ± 6,8 and the female‐to‐male ratio was 1.9.

### 2.8 Statistical analysis

Due to the limited number of samples data did not show normal distribution therefore, the nonparametric Kruskal–Wallis test was applied to cell proliferation and migration data. The normality of the data was assessed by the Shapiro–Wilk test. Analyses for patient‐derived oligodendroglioma (PD‐OG) and glioblastoma (PD‐GBM) cells were also compared for the different time points with multiple *t*‐tests. However, due to the limited number of samples in studies with primary cells, data was presented without statistical interpretation. Gene expression analysis data to compare IDH wild type and IDH mutant patient samples were analysed with unpaired *t*‐test. Finally, WHO grade comparison of qPCR results was done by Kruskal–Wallis test. All statistical analyses were done with GraphPad Prism 9 software. Statistical analysis results were shared as Figure S1.

No prior sample size calculation was performed for this study. It can be noted as a limitation of this study. In all cell culture studies with both cell lines and primary cells, the experimenter was not blind to the study groups. However statistical data were analysed blindly. For qPCR and flow cytometry studies, the experimenter was blind to all groups.

## 3 RESULTS

### 3.1 Silencing of the MEG3 gene inhibited the proliferation of glioma cells whereas MEG3 overexpression promoted cell proliferation

**FIGURE 1:**
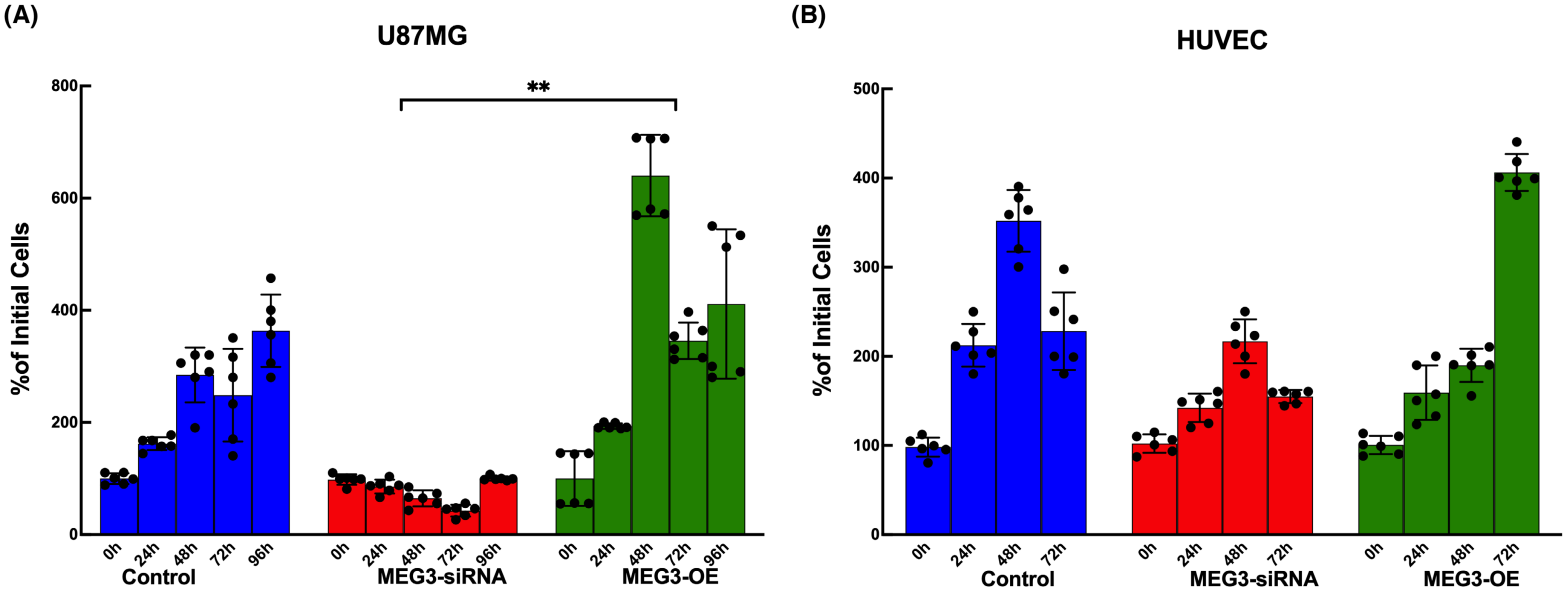
Cell proliferation assay in MEG3 inhibited and overexpressed cells. (A) U87MG (B) HUVEC cells. The assay was conducted for 96 h in glioma and 72 h in HUVEC cells. (*n* = 6 for each group), **p < 0.01.

MEG3 silenced and overexpressed in non‐cancerous HUVEC cells and U87MG glioma cells were compared to untreated control cells. U87MG glioma cells showed reduced cell proliferation when MEG3 was silenced. Whereas, cell proliferation was enhanced in these cells when the MEG3 gene was overexpressed for 96 h (*p* = 0.012) (Figure 1A). On the other hand, among the three groups of MEG3 silenced and overexpressed HUVEC cells, which were incubated for either 24, 48 or 72 h, only the 72 h group showed significant change in cell proliferation (Figure 1B) and there was no significant change between overall groups. The cell proliferation and viability analysis of HUVEC cells were done until 72 h after seeding whereas this was done until 96 h in U87MG cells. This difference in the time frame for analysis was due to HUVEC cells showing more proliferation potential which limits the amount of time needed for cell proliferation as a result of the contact inhibition of cells.

### 3.2 MEG3 gene silencing reduced the migration of both U87MG glioma and HUVEC cells

**FIGURE 2:**
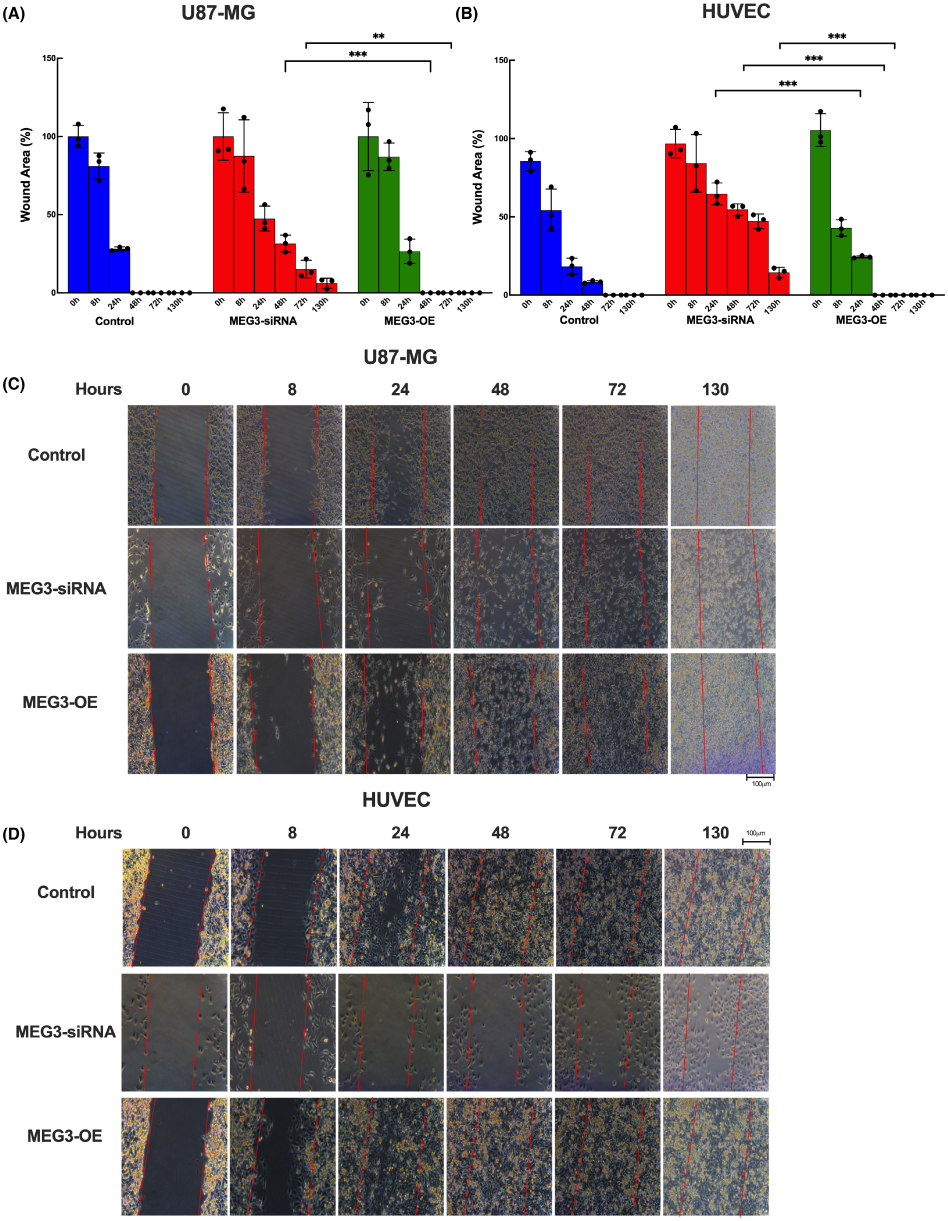
Cell migration assay in MEG3 inhibited and overexpressed cells (A) U87MG (B) HUVEC cells. Migration of cells to the wounded area has been photographed at 0, 8, 24, 48, 72 and 130 h in both cells. Microscope photographs were taken at 40× magnification. Bars indicate 100 μm length. (*n* = 6 for each group), **p < 0.01, ***p < 0.001.

To understand the effect of the MEG3 gene on cell migration, a wound‐healing assay was performed. Silencing of the MEG3 gene significantly reduced cell migration in U87MG glioma at 48 (*p* < 0.001) and 72 (*p* = 0.010) hours of analysis (Figure 2A) and at 24 (*p* < 0.001), 48 (*p* < 0.001) and 72 (*p* < 0.001) hours of analysis in HUVEC cells (Figure 2B). As for the MEG3 overexpression group, their cell migration was not significantly different from that of the non‐transfected cells. Cell migration was observed for up to 130 h following wound formation. MEG3 gene overexpressing cells re‐covered the wounded area within 48 h in both cell types whereas MEG3 silenced cells could not fully recover the wounded area even after 130 h upon scratch formation (Figure 2C). Moreover, MEG3 silencing caused slower cell migration in HUVEC cells compared to U87MG cells.

### 3.3 MEG3 gene silencing improved chemosensitivity for 5FU in glioma cells

**FIGURE 3:**
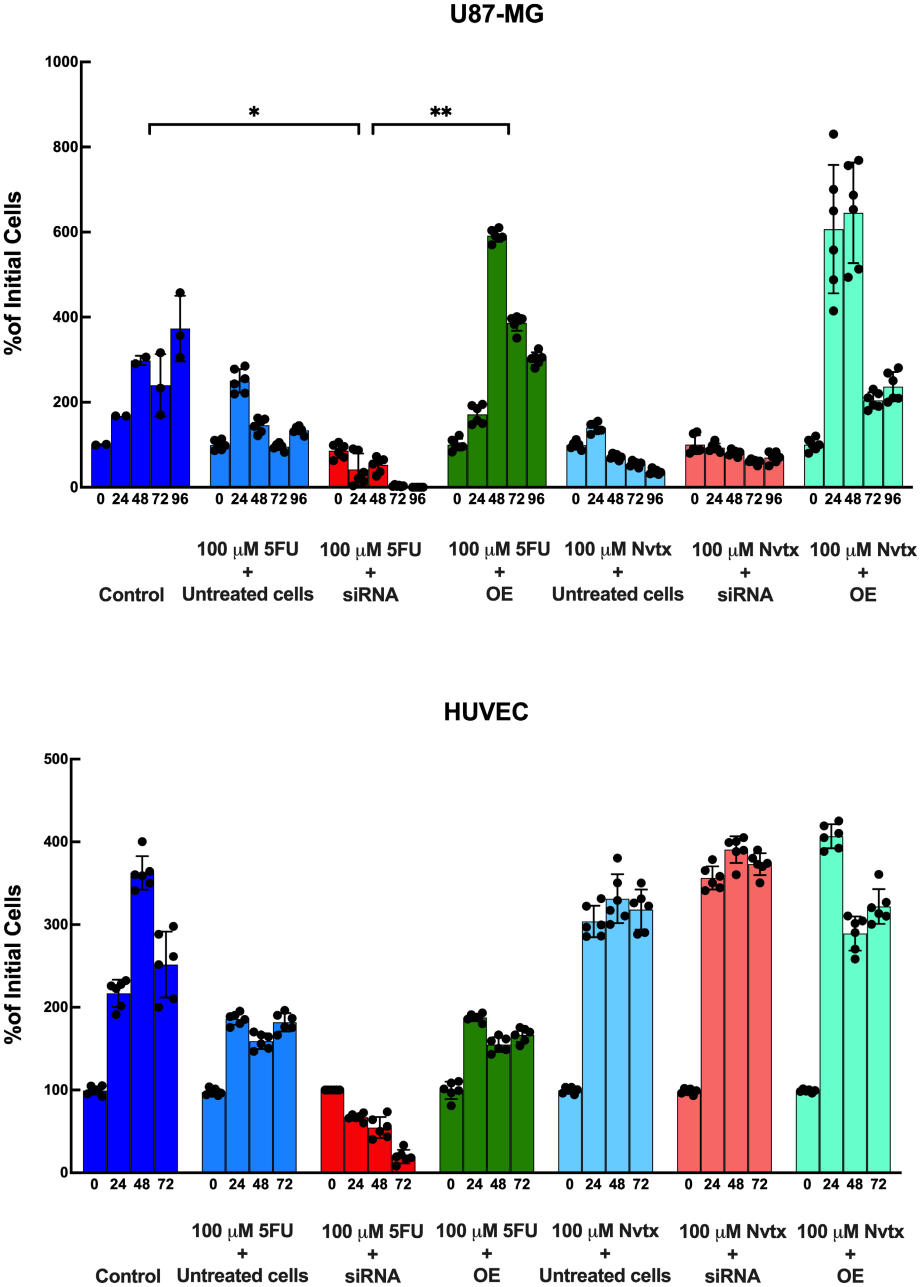
Chemosensitivity assay with 5‐fluorouracil and navitoclax (A) U87MG and (B) HUVEC cells. Assay was conducted for 96 h in glioma and 72 h in HUVEC cells. (*n* = 6 for each group), *p < 0.05, **p < 0.01.

The role of the MEG3 gene in the chemosensitivity of cells to two different drugs with different mechanisms of action was evaluated by the administration of these drugs and the assessment of cell viability. Although the 5FU treatment had a minor negative effect on U87MG glioma cell viability in nontransfected control cells expressing normal levels of MEG3, did not significantly inhibit cell proliferation. However, when MEG3 was suppressed, cells immediately died after 24 h of treatment and there was no resistant cell proliferation after 72 h of incubation (*p* = 0.0003). On the other hand, MEG3 overexpression significantly increased the proliferation of U87MG cells despite the cells being treated with 100 μM 5FU (*p* = 0.0311) (Figure 3A). We tested different concentrations of temozolomide activity on both MEG3‐inhibited and overexpressed U87 cells. The 200 μM concentration of temozolomide did not significantly inhibit cell proliferation in both MEG3‐inhibited and overexpressed U87 cells. On the other hand, incorporation of temozolamide with MEG3 siRNA triggered cell death, while U87 overexpression had no significant effect on cell proliferation (Appendix S1). The effects of MEG3 silencing on HUVEC cells were almost the same as it was on glioma cells. However, the overexpression of MEG3 did not significantly improve cell viability in the transfected cells compared to the untreated HUVEC cells (Figure 3B).

The effects of Nvtx were also tested. The Nvtx treatment did not significantly inhibit the viability of glioma cells expressing MEG3 at normal levels. As for the transfected cells, neither suppression nor the overexpression of the MEG3 gene showed significant inhibition of cell proliferation. However, MEG3 overexpression significantly promoted cell proliferation within 24 and 48 h of treatment in U87MG glioma cells (Figure 3A). HUVEC cells were also not affected by MEG3 activity when treated with Nvtx. Neither an increase nor a decrease in cell viability was observed in the MEG3 gene silenced and MEG3 overexpressed HUVEC cells treated with Nvtx (Figure 3B). Hence, we can propose that MEG3 activity is important in the chemosensitivity of glioma cells to 5FU but not to Nvtx. This selective activity may be due to the MEG3 gene's interactions with the selected drug mechanism of action, DNA synthesis.

### 3.4 MEG3 may have different roles in different glioma cells and pathological subtypes of glioma tumours

**FIGURE 4:**
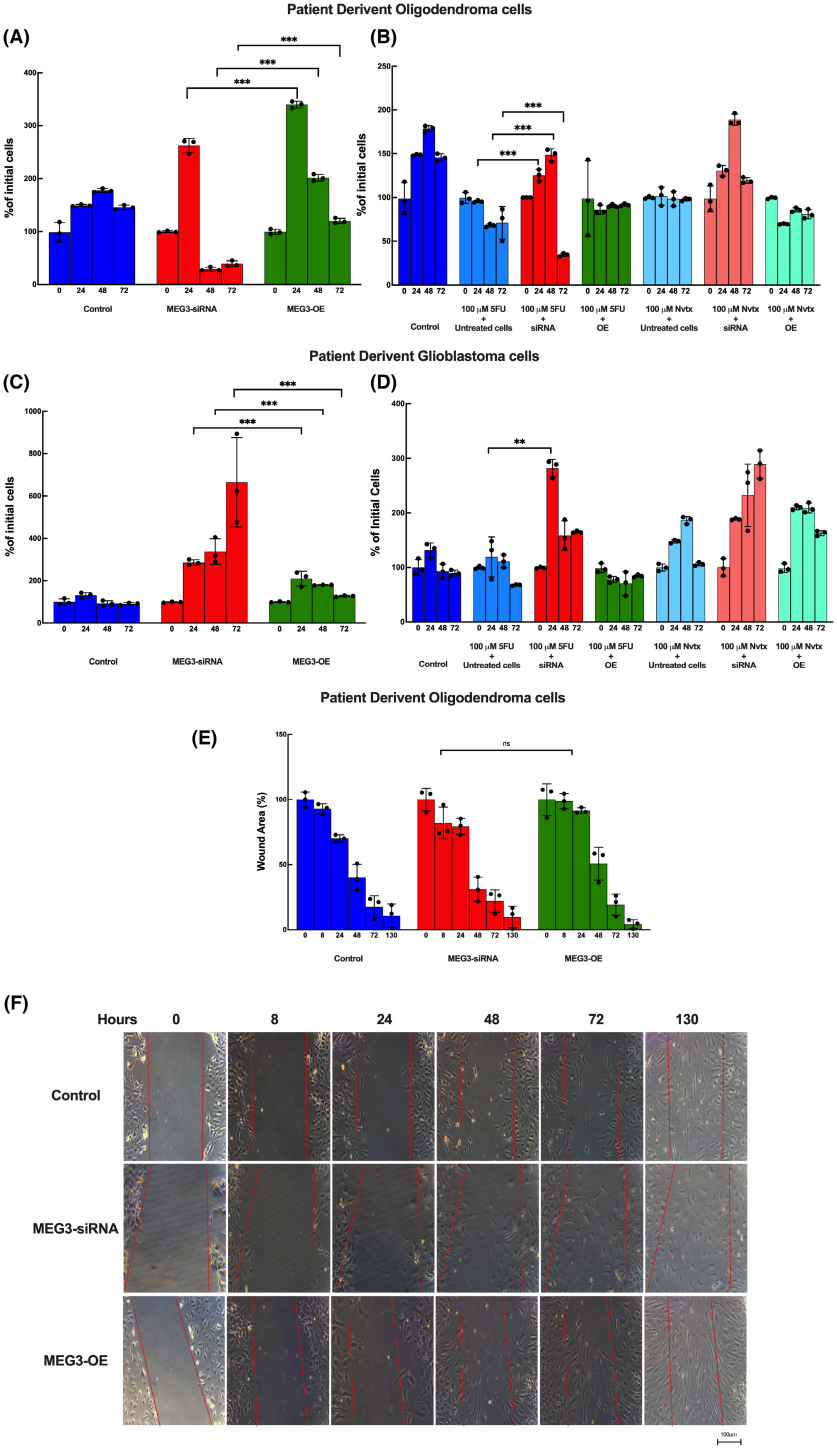
Cell proliferation (A, C), chemosensitivity (B, D), and cell migration (E) assay of MEG3 inhibited, overexpressed, patient‐derived oligodendroglioma and patient‐derived glioblastoma cells. Migration of cells to the wounded area has been photographed at 0, 8, 24, 48, 72 and 130 h in both cells. (*n* = 3 for each group). Microscope photographs were taken at 40× magnification. Bars indicate 100 μm length. **p < 0.01, ***p < 0.001, ns, not significant.

Patient‐derived glioma tumour samples were used to assess the effects of the MEG3 gene on the cell viability, chemosensitivity and cell migration of glioma cells. Oligodendroglial (PD‐OG) and glioblastoma (PD‐GBM) cells were obtained from oligodendroglioma and glioblastoma patient's tumours, respectively, and the cells were subjected to the viability, migration and chemosensitivity assays as the other cell lines. Due to the limited number of samples, primary cell studies were represented without statistical comparisons. However, suppression of the MEG3 gene decreased the viability of PD‐OG cells at the 48th and 72nd hours whereas PD‐GBM cell viability was promoted upon MEG3 silencing. On the other hand, overexpression of MEG3 did not affect cell viability in either of these cell types (Figure 4A,C). The drug responses of patient‐derived cells also differed between different glioma cells. MEG3 silenced PD‐OG cells treated with 5FU showed a reduction in cell viability whereas PD‐GBM cells showed less cell viability at 48 and 72 h upon treatment. The MEG3 silenced and MEG3 overexpressed cells that were treated with Nvtx did not show any change in cell viability (Figure 4B,D). The migration potential of PD‐OG cells was not affected by MEG3 activity. Neither silenced nor overexpressed MEG3 oligodendroglial cells showed any change in cell migration (Figure 4E,F). PD‐GBM cells were also not affected by MEG3 activity (Data not shown). Although both cell lines originated from glioma tumours they were differently affected by MEG3 expression with respect to cell viability and 5FU response, indicating the different roles of MEG3 in different types of glioma cells.

**FIGURE 5:**
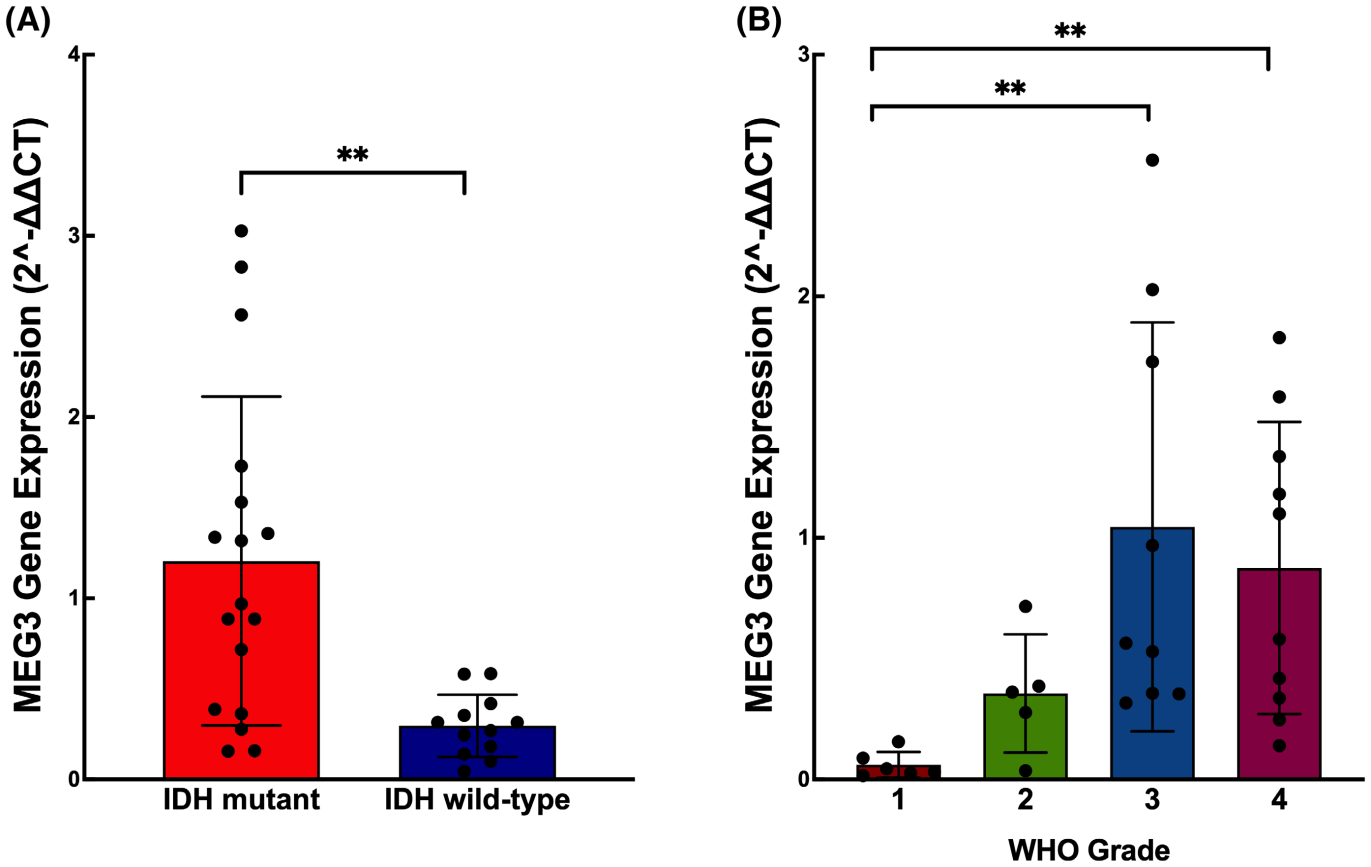
qPCR results of MEG3 gene in a) IDH mutant and IDH wild‐type glioma cells. b) MEG3 expression in glioma tumours with different WHO grades. (*n* = 30) **p < 0.001.

MEG3 gene expression levels were analysed in patient‐derived glioma tumours including different histopathological subtypes, grades, and IDH statuses. RNAs were derived from 30 patients. The level of MEG3 gene expression was significantly higher in IDH mutant glioma tumours compared to IDH wild‐type glioma (*p* = 0.0021) (Figure 5A). Moreover, the level of MEG3 had a partial correlation with the tumour grade. Grade 3 (*p* = 0.003) and Grade 4 (*p* = 0.0061) tumours showed a significantly increased level of MEG3 gene expression whereas Grade 1 and Grade 2 tumours showed the least amount of expression (Figure 5B). It might be speculated that the MEG3 overexpression and siRNA transfections were less impactful on GBM cells due to the already low levels of MEG3 expression in Grade 4 tumour cells. Similarly, the fact that MEG3 expression is higher in Grade 3 patients might be causing a greater difference between MEG3 overexpressed or siRNA treated oligodendroglioma cells and their controls. Additionally, the finding that MEG3's expression is higher in IDH mutant patients than wild‐type patients is consistent with the tumour suppressor role of the MEG3 gene demonstrated in the previous studies.

### 3.5 Suppression of MEG3 induces apoptotic cell death

**FIGURE 6:**
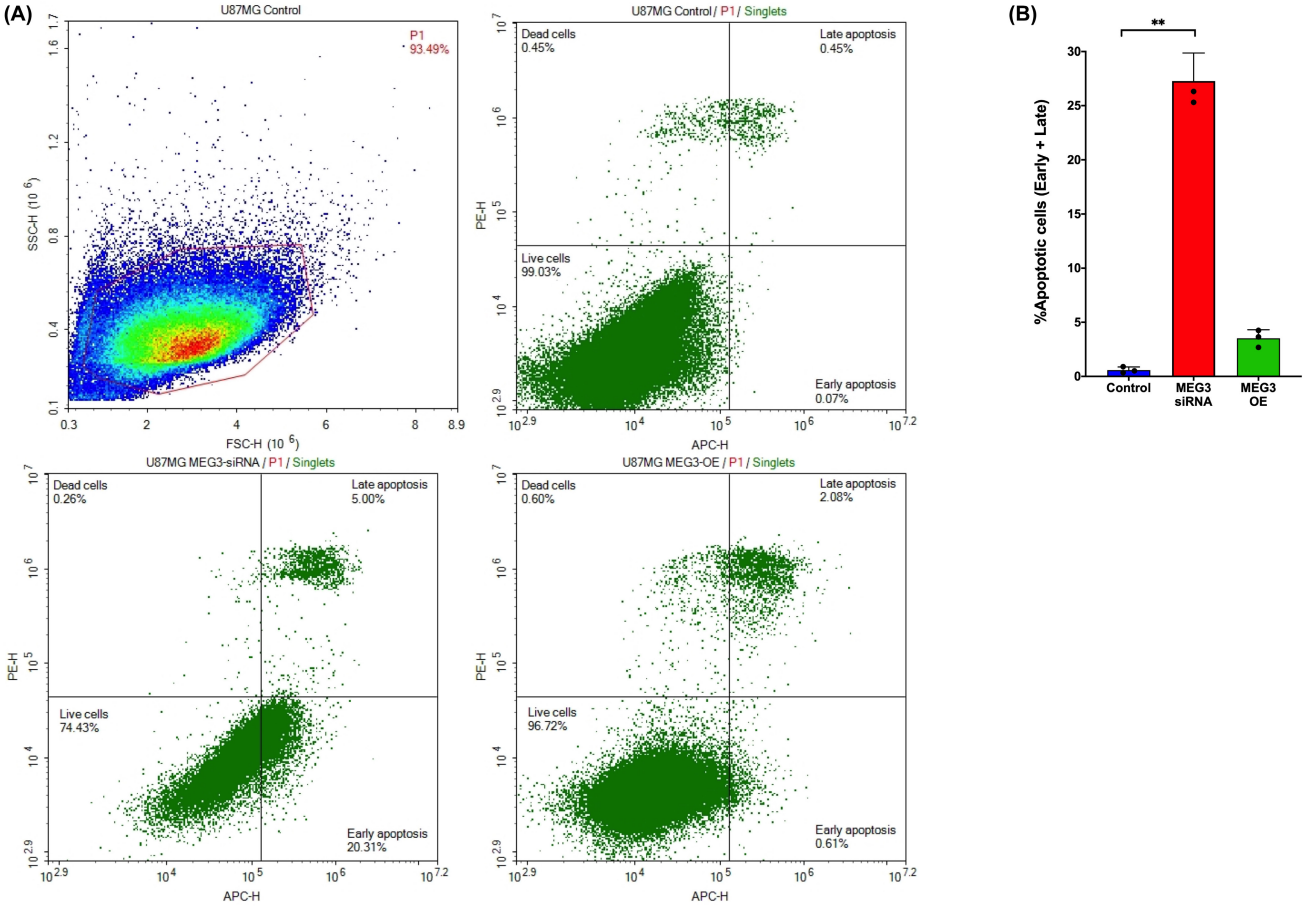
Evaluation of cell death. (A) Flow cytometry dot plot (B) Comparison of groups. (*n* = 3 for each group) **p < 0.001.

The MEG3 associated cell death mechanism of action was investigated by evaluating the cell death in U87MG glioma cells. MEG3 silencing in glioma cells switched the cell populations into early apoptotic and late apoptotic cell phenotypes, whereas the control and MEG3 overexpressed cells showed no significant apoptotic induction (Figure 6A). In the MEG3 silenced glioma group, 25% of the cells were engaged in a cell death process (*p* = 0.0036) while only 0.5% and 2% of control and MEG3 overexpressing cells were, respectively, undergoing cell death (Figure 6B).

## 4 DISCUSSION

The study presented here demonstrates a well‐defined characterisation of how long non‐coding RNA (lncRNA) MEG3 gene plays different roles in different types of glioma. Cell proliferation, cell migration, and drug sensitivity may vary between different glioma subtypes when MEG3 is silenced and overexpressed in glioma cells. We proposed a model experimental study that explores the use of available chemotherapeutics by modulating the MEG3 gene.

Glial tumours are a heterogeneous group of tumour categories that include various molecular signatures that form distinct types of tumours. Recent shreds of evidence confirmed that long noncoding RNAs (LncRNAs) are modulating tumorigenesis for various cancer types. Therefore, exploring the role of LncRNAs in glioma can facilitate our understanding of its development. MEG3 is an imprinted gene located at the chromosome 14q and expressed in various tissue types. MEG3 has a tumour suppressor role in normal tissues and its loss of function has been associated with various cancer types including bladder, breast, bone marrow, cervix, colon, liver lung and meninges.^12^ Loss of MEG3 activity in these different cancer types is attributed to aberrant DNA methylation.^13^, ^14^

The level of MEG3 expression in glial cells has been investigated in different studies with patient‐derived tissues and various cell lines. Studies including glioma tumours and controls reported that MEG3 expression level was decreased in glioma tissues compared to the non‐cancerous glial cells of the patients'.^15^, ^16^, ^17^ In vitro studies including the U251, U87 and A172 cell lines have also reported that MEG3 gene expression was relatively downregulated in these cell lines compared to the control cells.^18^, ^19^ Furthermore, Tong et al. showed that MEG3 suppressed the proliferation, migration and invasion of glioma cells^17^ whereas, Gong et al.^17^ reported that MEG3 suppressed glioma cell proliferation and induced cell cycle progression, overexpression of MEG3 which weakened the Wnt/β catenin pathway.

Considering the results of these previous studies, we conducted a comparative analysis of MEG3 activity in glioma and non‐cancerous HUVEC cells by suppressing and overexpressing this gene. Interestingly, we found out that the suppression of MEG3 limited the proliferation of U87 cells while it promoted cell proliferation in the HUVEC cells. As for the overexpression of MEG3, it promoted cell proliferation in both U87 and HUVEC cells. Consistent with cell proliferation, cell migration was also suppressed when MEG3 was downregulated in U87 and HUVEC cells and it did not significantly change when MEG3 was overexpressed in both cell types. Although experiments were repeated at least two times the results were completely inconsistent with previously reported data on MEG3. Therefore, we suspected the U87 cells that we were experimenting on and decided to conduct the same experiments on patient‐derived glioma cells. Primary glioma cells were obtained from oligodendroglioma (PD‐OG) and glioblastoma patients' (PD‐GBM) cells. Upon the suppression of the MEG3 gene, PD‐OG cells showed reduced cell proliferation and cell migration similar to the U87MG cells, whereas the PD‐GBM cells demonstrated increased cell proliferation and migration. In other words, the PD‐OG results did not fit the previously acquired data on MEG3 activity but the PD‐GBM results were consistent with them. Therefore, we proposed that MEG3 activity depends on the cell's context and the cell type the gene is found in. This hypothesis was also partially formed considering the epigenetic differences between cells which have previously been drawn attention to by several studies.^19^, ^20^, ^21^

Based on our hypothesis that cells can be affected in different ways by MEG3 activity, we evaluated how MEG3 gene expression impacts drug sensitivity in glioma cells. Ma et al.^22^ previously showed that the overexpression of MEG3 enhanced the chemosensitivity of U87MG cells to cisplatin whereas the suppression of MEG3 increased the cells' resistance to the same drug. Here, we wanted to evaluate the effects of two different drugs with different mechanisms of action on glioma cells. 5FU and Nvtx were chosen for increasing the range of drugs available for glioma treatment as none of these drugs are currently used for this purpose. However, there are many experimental studies indicating their partial efficacy in suppressing tumour growth and cell proliferation.^23^, ^24^, ^25^, ^26^ Silencing of MEG3 in glial and HUVEC cells that have been treated with 5FU, caused cell proliferation in these cells to become inhibited. Whereas, the MEG3 downregulated cells that have been treated with Nvtx showed no significant change in their proliferation.

In patient‐derived oligodendroglioma cells 5FU significantly decreased cell viability when MEG3 was suppressed. However, Nvtx did not induce any change in cell viability when MEG3 was suppressed. Neither 5FU nor Nvtx was found to be effective in reducing the cell viability of MEG3 suppressed and overexpressed patient derived glioblastoma cells. The suppression of MEG3 enhanced the sensitivity of oligodendroglioma cells to the 5FU drug. A previous study by Li et al.^27^ reported that the overexpression of MEG3 promoted chemosensitivity to oxaliplatin. This result was consistent with the activity of cisplatin demonstrated in a study done by Ma et al.^22^ Our results are opposing with the conclusions made by these studies in that higher levels of chemosensitivity were achieved when MEG3 was suppressed rather than overexpressed. However, this may be due to the abundant expression of MEG3 in the cells we worked on and tested for chemosensitivity. Therefore, we can again propose that the activity of MEG3 is specific to the cell type and the cellular context, which dictate chemosensitivity.

MEG3 activity differences between different cell lines pushed us to pose this question: Are there any significant differences between patient, disease and tumour‐associated characteristics of various glioma types? It has been reported that MEG3 gene expression levels were lower in glioma samples compared to normal and para‐carcinogenic samples taken from patients. High‐grade (Grade 3‐4) glioma tumours showed decreased expression of MEG3 compared to low‐grade glioma (Grade 1‐2) tumours.^16^, ^17^, ^19^ There is a significant correlation between MEG3 expression and overall survival.^17^ Furthermore, studies have shown that lower levels of MEG3 expression are associated with a higher WHO grade of tumour, older age at the time of diagnosis, low Karnofsky performance score (KPS), the presence of the wild‐type isocitrate dehydrogenase (IDH), tumour recurrence and poor overall survival.^7^ Although there is a significant reverse correlation between tumour malignancy and MEG3 expression in previous reports, we found that MEG3 activity differs between different cell lines and is mostly correlated with the IDH1 mutation status. IDH1 mutant tumours showed significant overexpression of MEG3. The maximum MEG3 expression was observed in Grade 3 tumours indicating the contribution of IDH1 mutations since IDH1 mutant gliomas are the most frequently observed among all the grades of glioma tumours.^28^

Finally, MEG3 suppression was found to possibly induce apoptotic cell death. Previous studies showed that MEG3 overexpression would induce apoptosis in lung carcinoma and glioma cells.^18^, ^29^ Contrary to these findings, our experiments revealed that the induction of cell death was observed when MEG3 was suppressed. Other studies showed that when MEG3 has overexpressed in U251 and U87MG cells the frequency of dead cells were at somewhere between 12% and 15%. This meant that only a minor group of cells were affected by MEG3 overexpression.^18^ In our study, we reported that 25% of MEG3 downregulated and 2.5% of MEG3 overexpressed cells underwent cell death. In both, our study and previous studies, only a minor fraction of the total cell showed apoptosis indicating a more complicated role of MEG3 in cell death due to its heterogeneous expression in different cells.

## 5 CONCLUSIONS

Despite various studies indicating that the loss of MEG3 expression was detected in various cancer types including glioma, here we report that different glioma cells may be affected differently by MEG3 expression and MEG3 manipulation. U87MG glioma cells and patient derived OLG cells showed that when MEG3 is post‐transcriptionally suppressed cell proliferation, and migration in glioma cells are reduced and chemosensitivity to the 5FU drug is achieved. This sensitivity to the 5FU drug upon MEG3 suppression was not observed in glioblastoma and endothelial HUVEC cells. Here, we propose that the MEG3 lncRNA plays a complicated role that changes for different cancer types. As a take home message from this study, the role of MEG3 lncRNA cancer cells requires further attention to be fully understood.

## AUTHOR CONTRIBUTIONS

**Zehra Degirmenci:** Conceptualization (equal); investigation (equal); methodology (equal); writing – original draft (equal). **Sena Unver:** Data curation (equal); formal analysis (equal); methodology (equal). **Turker Kilic:** Funding acquisition (equal); project administration (equal); resources (equal). **Timucin Avsar:** Conceptualization (equal); data curation (equal); funding acquisition (lead); project administration (equal); supervision (lead); writing – original draft (lead).

## FUNDING INFORMATION

The study is funded by Bahcesehir University (BAU) Scientific Research Project Council (Project Number: BAP.2020 – 02.19).

## CONFLICT OF INTEREST STATEMENT

The authors confirm that there are no conflicts of interest.

## Supporting information


Figure S1

Appendix S1

## Data Availability

All data produced by this study can be provided upon reasonable request from the corresponding author.
