# Supplementary material for: Silencing of the MEG3 gene promoted anti‐cancer activity and drug sensitivity in glioma
Source: J Cell Mol Med. 2023 Jul 31;27(17):2603–13. doi: 10.1111/jcmm.17883 (PMC10468657; doi:10.1111/jcmm.17883)

Supplementary File – 1:
MEG3 gene expression in databases

The Cancer Genome Atlas (TCGA), Cell 2013
Glioblastoma

a)

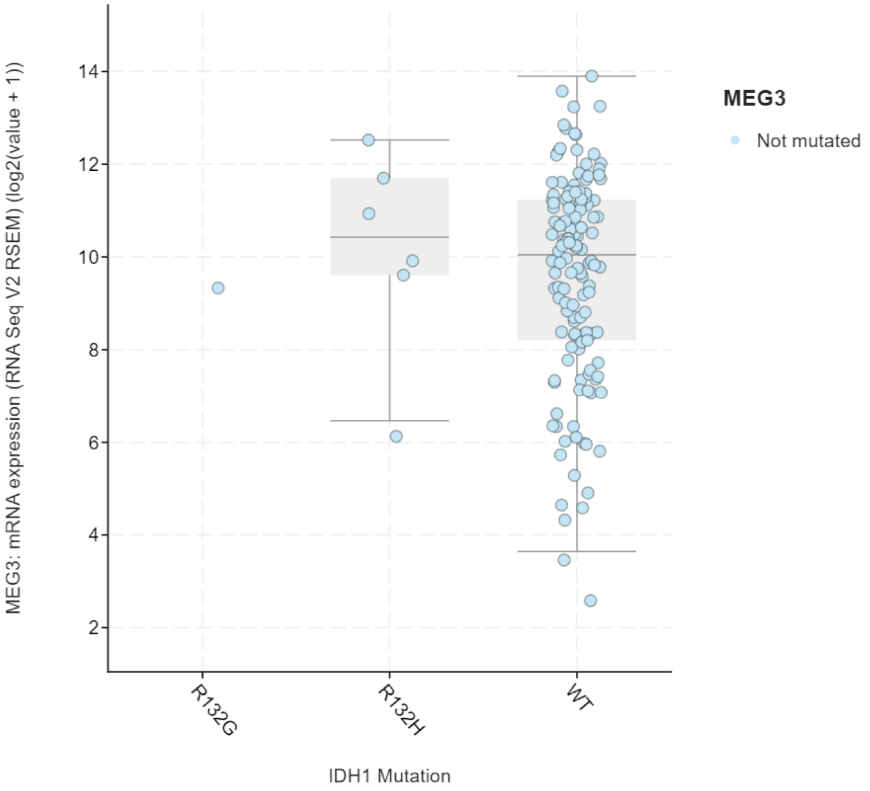

b)

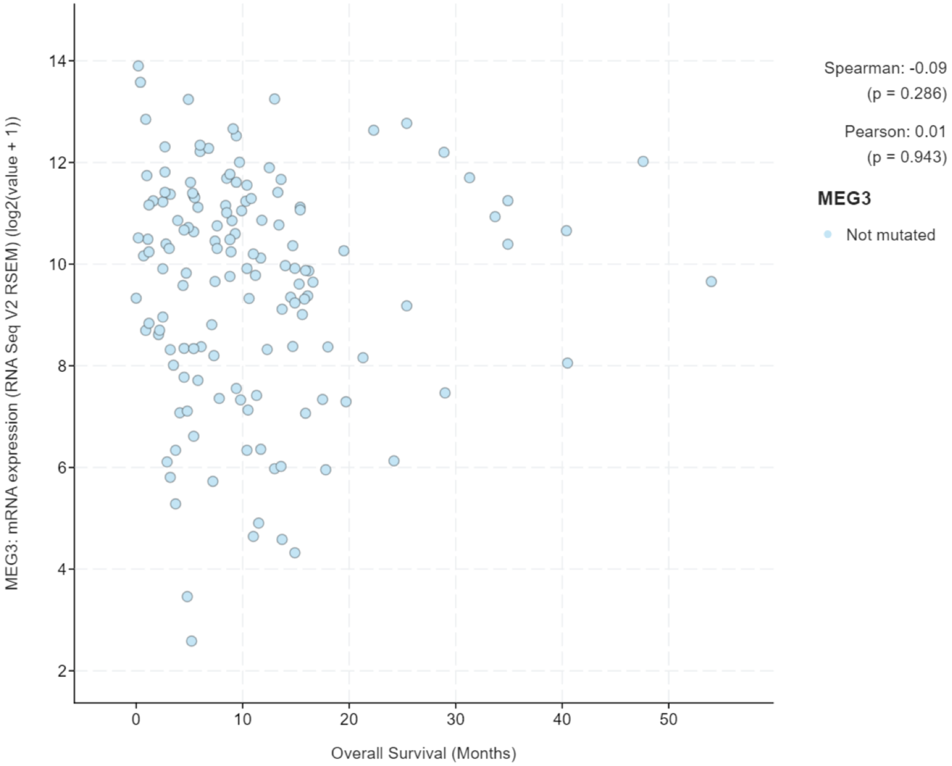

TCGA GDAC Firehose Legacy Glioblastoma

c)

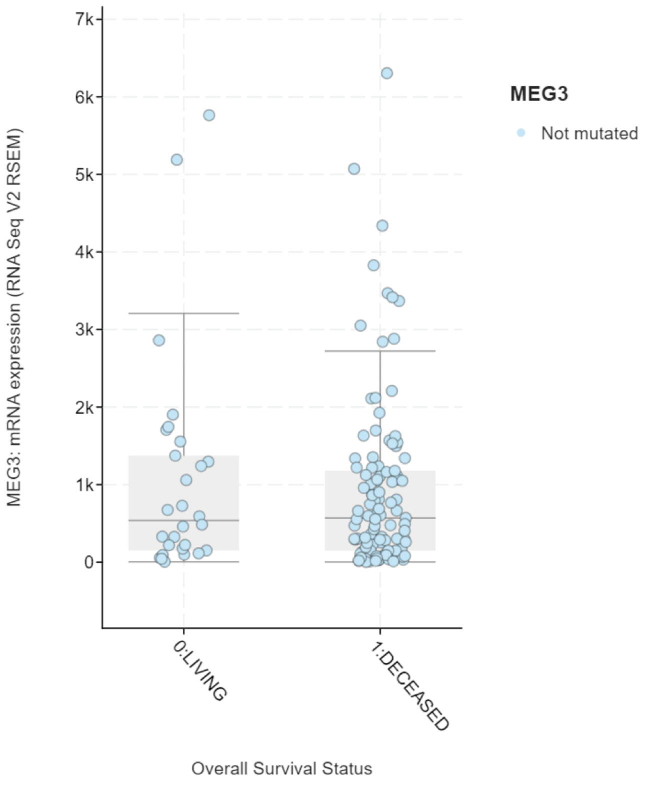

d)

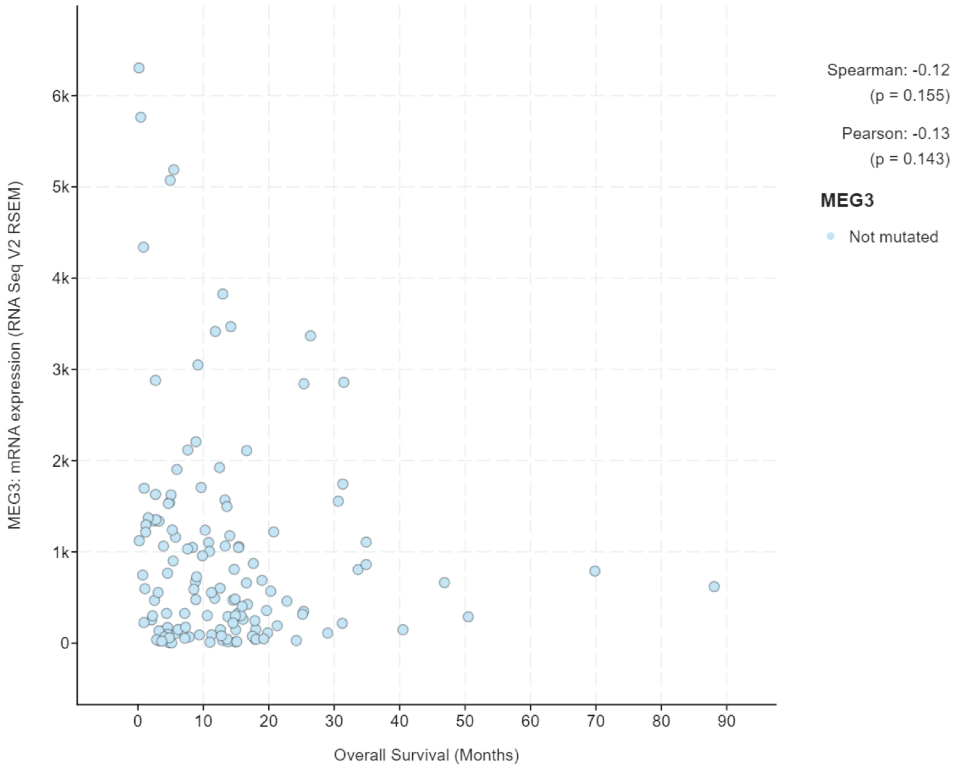

TCGA PanCancer Atlas
Glioblastoma

e)

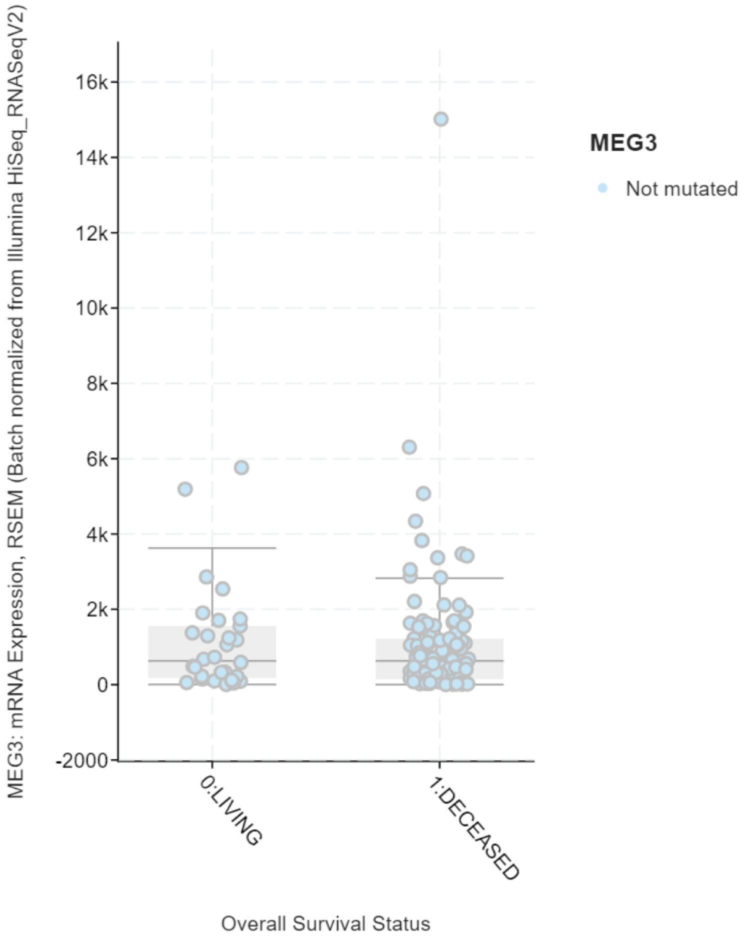

f)

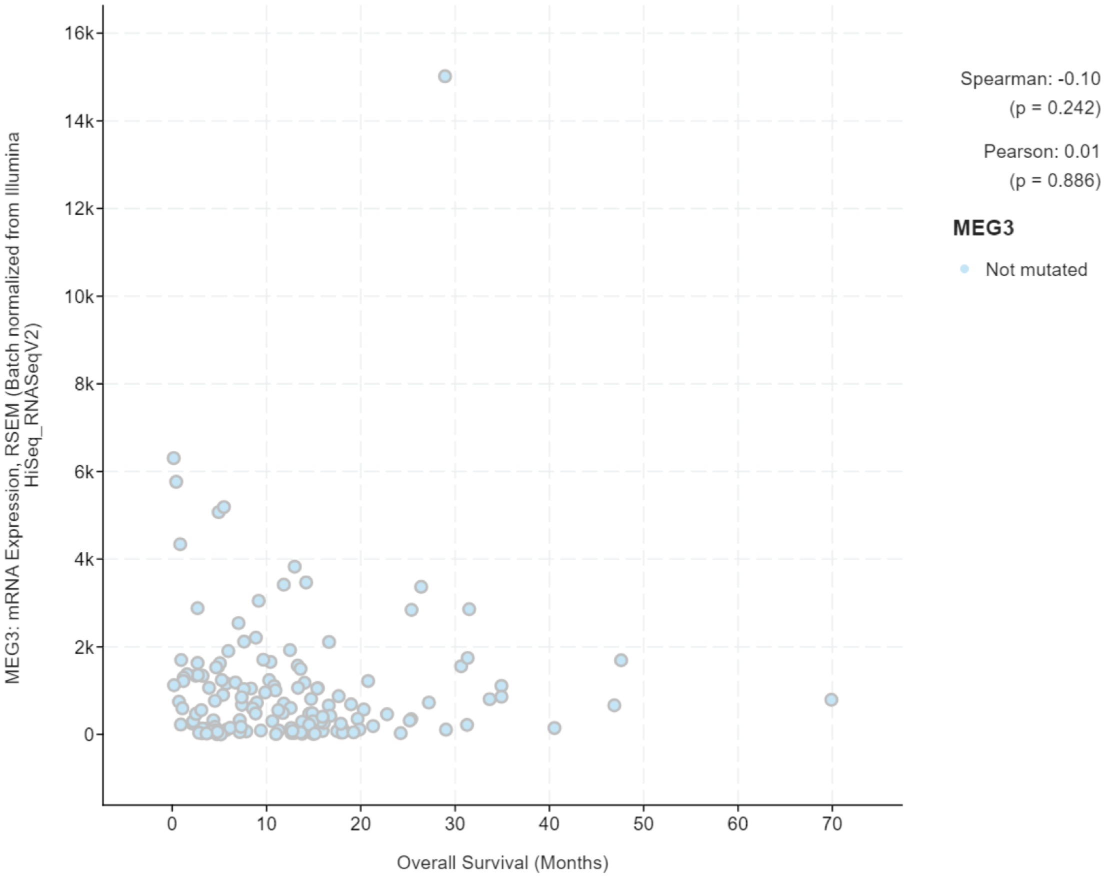

GDAC Firehose Legacy
Lower Grade Glioma

g)

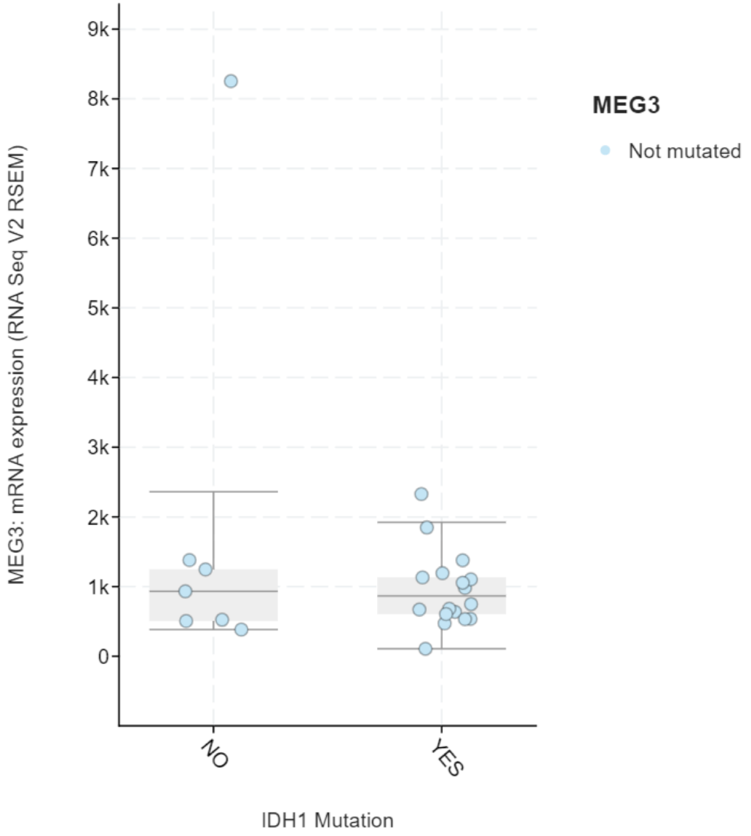

h)

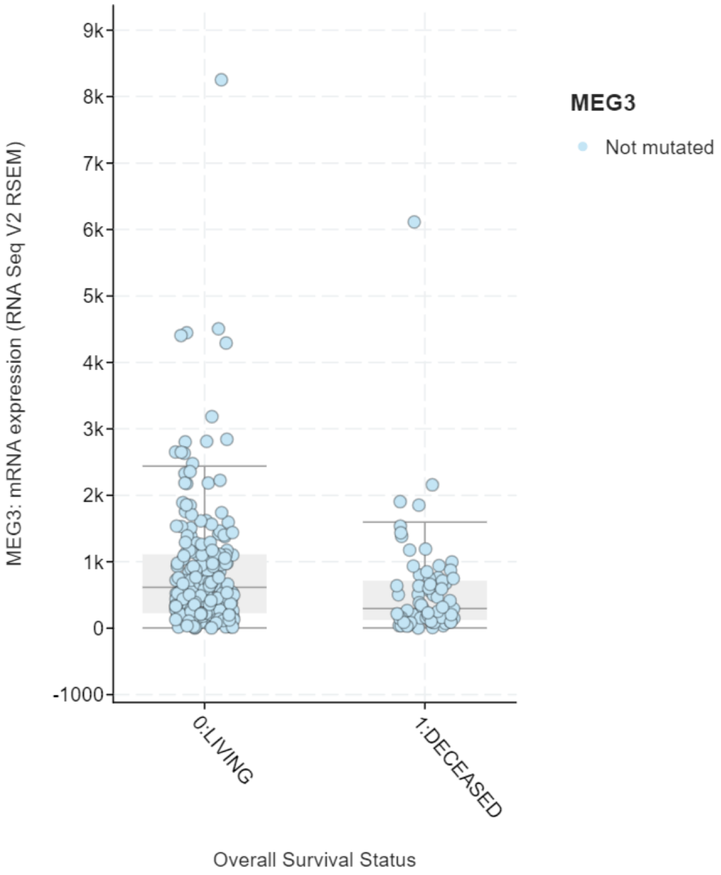

i)

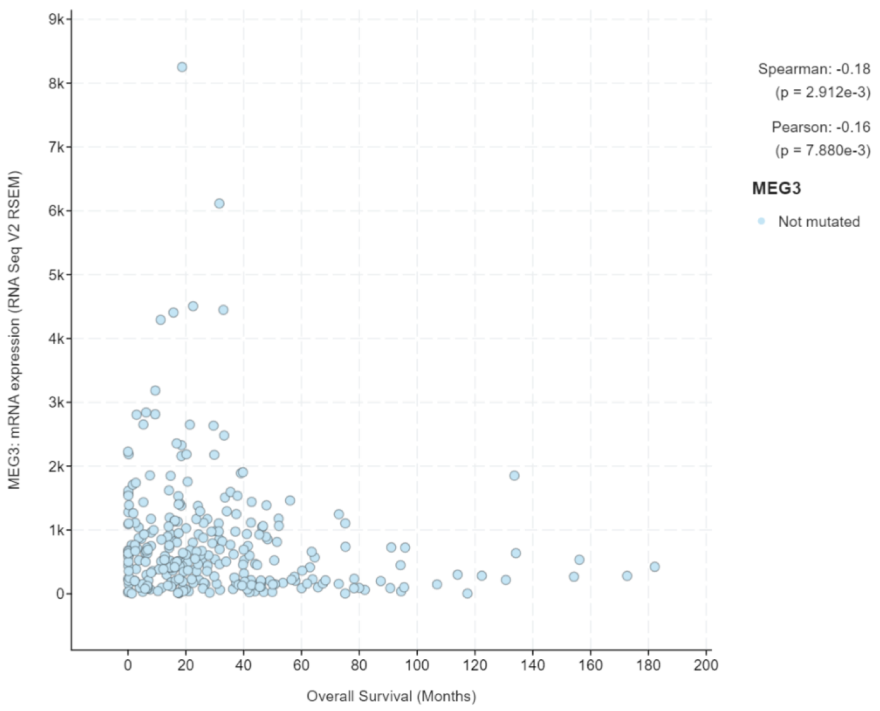

TCGA PanCancer Atlas
Lower Grade Glioma

j)

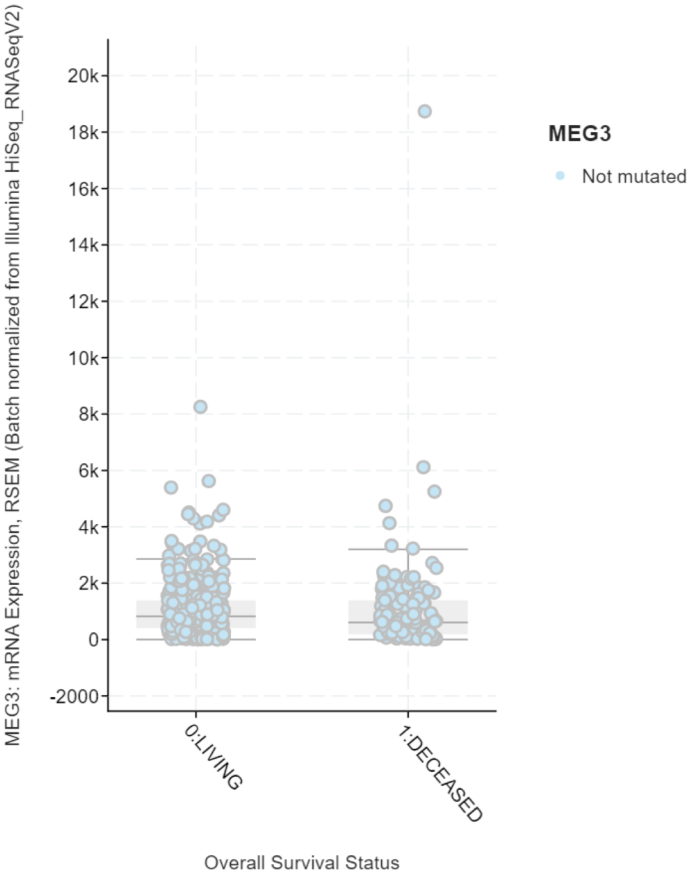

k)

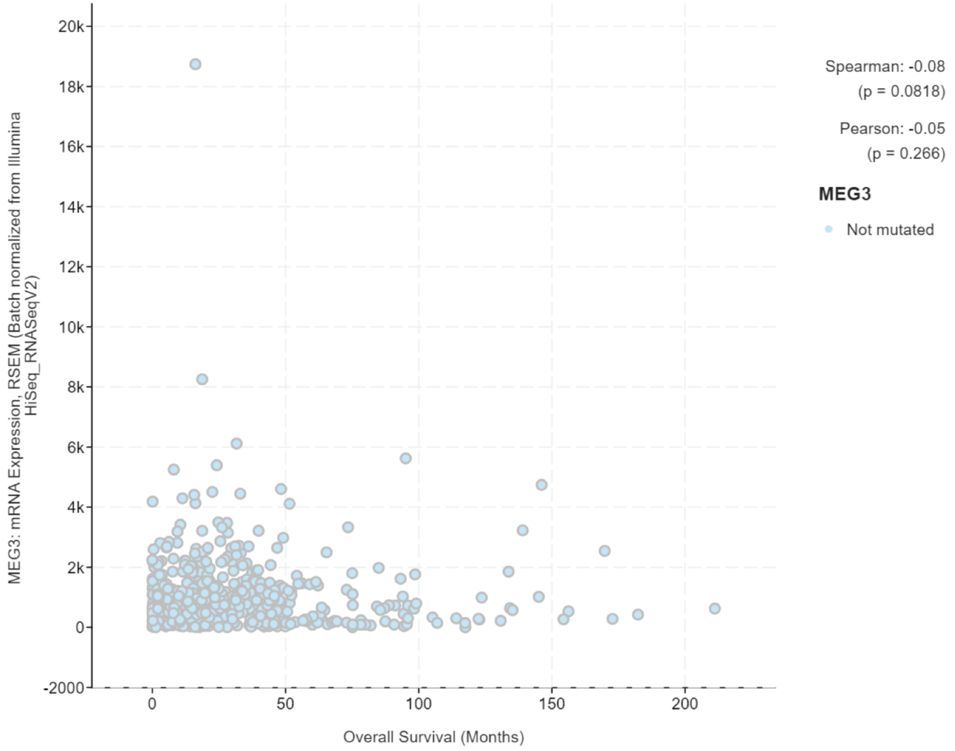

TCGA PanCancer Atlas
Lower Grade Glioma

l)

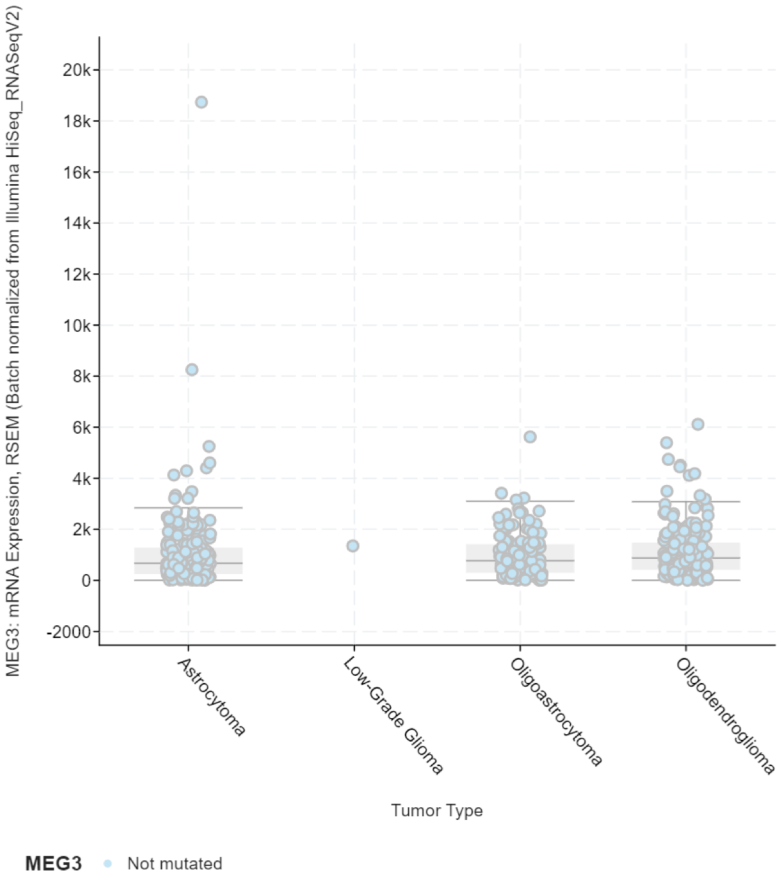

m)

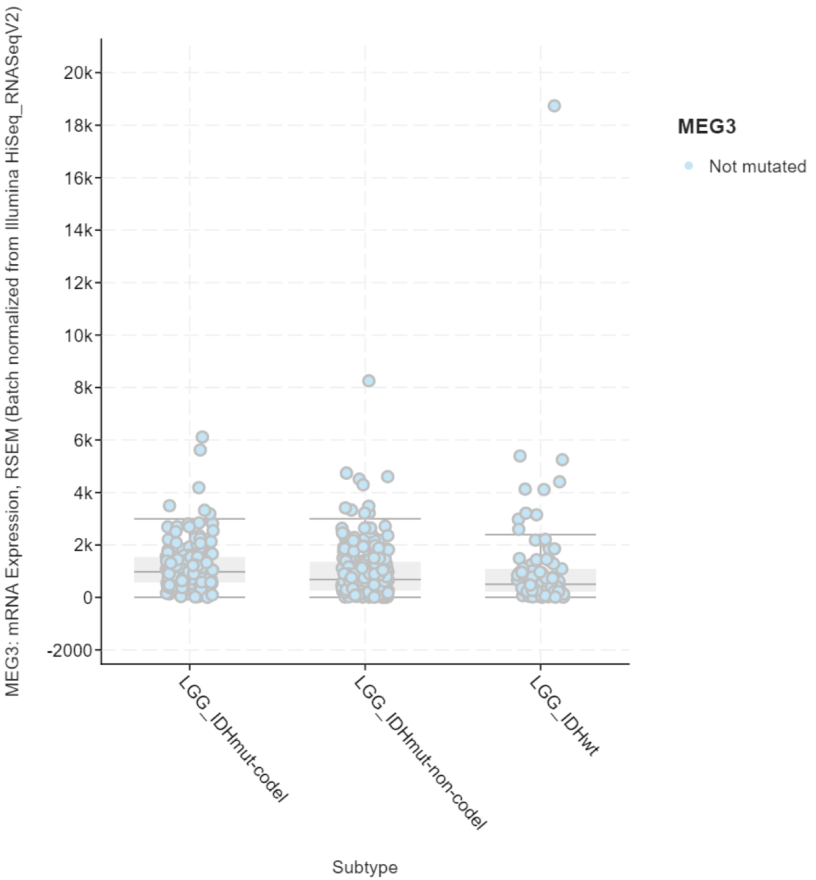

Supplement: Supplementary file 2 — Appendix S1 [file JCMM-27-2603-s002.pdf]
